# 2,3-Bis(2-methoxy­phen­yl)tetra­zolium-5-thiol­ate–acetone–dichloro­methane (1/0.4/0.1)

**DOI:** 10.1107/S1600536808039731

**Published:** 2008-12-03

**Authors:** Karel G. von Eschwege, Alfred Muller

**Affiliations:** a Department of Chemistry, University of the Free State, PO Box 339, Bloemfontein 9300, South Africa; b Department of Chemistry, University of Johannesburg (APK Campus), PO Box 524, Aucklandpark, Johannesburg 2006, South Africa

## Abstract

In the title compound, C_15_H_14_N_4_O_2_S·0.4C_3_H_6_O·0.1CH_2_Cl_2_, two benzene rings in the *ortho*-meth­oxy dehydro­dithizone (omd) mol­ecule are twisted out of the tetra­zole plane with the meth­oxy groups in a *cis* orientation relative to the tetrazole backbone. The acetone is located on a special position. The dihedral angles formed by the benzene rings with the central five-membered ring are 63.14 (8) and 57.06 (6)°. In the crystal structure, the relatively short distance of 3.886 (3) Å between the centroids of benzene rings from two neighbouring omd mol­ecules indicate π–π stacking inter­actions.

## Related literature

For general background, see: Al-Salihy & Freiser (1970 ▶); Irving (1977 ▶); Allen (2002 ▶). For details of the synthesis, see: Mirkhalaf *et al.* (1998 ▶); Irving *et al.* (1971 ▶).
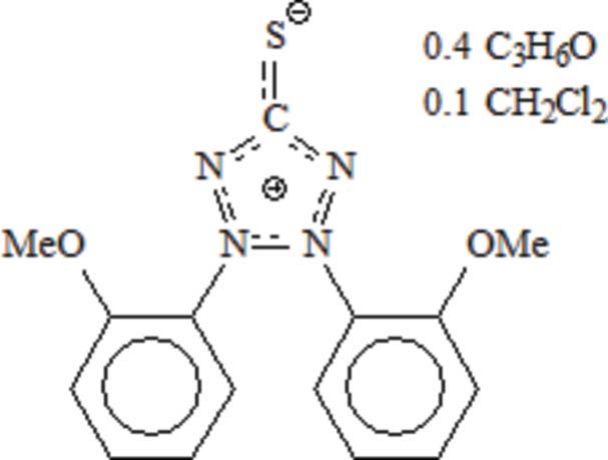


## Experimental

### 

#### Crystal data

C_15_H_14_N_4_O_2_S·0.4C_3_H_6_O·0.1CH_2_Cl_2_
*M* *_r_* = 346.09
Orthorhombic, 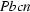

*a* = 19.5069 (13) Å
*b* = 12.5245 (7) Å
*c* = 13.2780 (10) Å
*V* = 3244.0 (4) Å^3^
*Z* = 8
Mo *K*α radiation
μ = 0.25 mm^−1^
*T* = 100 (2) K
0.33 × 0.12 × 0.11 mm

#### Data collection

Bruker X8 APEXII 4K Kappa CCD diffractometer
Absorption correction: multi-scan (*SADABS*; Bruker, 2008 ▶) *T*_min_ = 0.919, *T*_max_ = 0.972
10890 measured reflections
4013 independent reflections
2571 reflections with *I* > 2σ(*I*)
*R*_int_ = 0.051

#### Refinement

*R*[*F* ^2^ > 2σ(*F* ^2^)] = 0.059
*wR*(*F* ^2^) = 0.169
*S* = 1.06
4013 reflections
238 parameters
2 restraints
H-atom parameters constrained
Δρ_max_ = 0.83 e Å^−3^
Δρ_min_ = −0.57 e Å^−3^

### 

Data collection: *APEX2* (Bruker, 2008 ▶); cell refinement: *SAINT-Plus* (Bruker, 2004 ▶); data reduction: *SAINT-Plus* and *XPREP* (Bruker, 2004 ▶); program(s) used to solve structure: *SIR97* (Altomare *et al.*, 1999 ▶); program(s) used to refine structure: *SHELXL97* (Sheldrick, 2008 ▶); molecular graphics: *DIAMOND* (Brandenburg & Putz, 2005 ▶); software used to prepare material for publication: *WinGX* (Farrugia, 1999 ▶).

## Supplementary Material

Crystal structure: contains datablocks global, I. DOI: 10.1107/S1600536808039731/cv2466sup1.cif

Structure factors: contains datablocks I. DOI: 10.1107/S1600536808039731/cv2466Isup2.hkl

Additional supplementary materials:  crystallographic information; 3D view; checkCIF report

## Figures and Tables

**Fig. 1.**
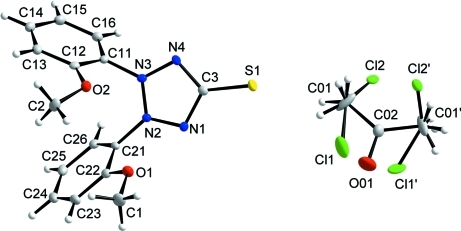
View of (2) with 30% probability displacement ellipsoids. Accented lettering indicate atoms generated by symmetry (2 - x, y, 3/2 - z).

**Fig. 2.**
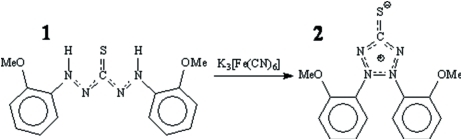
The formation of the title compound.

## supplementary crystallographic information

### Comment

The effect of electron donating (–CH_3_) and withdrawing groups (F, Cl, Br, I) on the phenyl rings of dithizone, (PhNHN)_2_CS, was investigated by Al-Salihy and Freiser (1970), and expressed in terms of acid dissociation constants. In view of dithizone's extensive applications in the field of heavy metals analyses (Irving, 1977) we have decided to execute an extended investigation of the above by, amongst others, also including methoxy groups substituted on the different phenyl ring positions. Growing suitable crystals for X-ray diffraction of the *ortho*-methoxy derivative of dithizone (1 on Scheme 2) proved to be problematic. However, oxidation of the same, resulting in the zwitter-ionic tetrazolium salt of the title compound, *ortho*-methoxy dehydrodithizone, (2), yielded a product that readily crystallizes in polar solvent mixtures. Herewith we present the crystal structure of the title compound (2).

In (2) (Fig. 1), all bond lengths and angles are normal (Allen, 2002). The phenyl rings adopt a non-parallel arrangement with the dehydrodithizone backbone with dihedral angles of 63.14 (8)° and 57.06 (6)° for rings C11—C16 and C21—C26 respectively, mainly due to their close proximities on the tetrazole moiety. The preferred orientation is supported by interaction of one of the methoxy moieties to N1 and the π-π stacking of the phenyl rings of C21—C26 situated around an inversion center (centroid to centroid distance = 3.886 Å, Table 1).

### Experimental

Reagents were purchased from Sigma-Aldrich, and solvents (AR) from Merck, and used without further purification. The *ortho*-methoxy derivative of dithizone, (*o*-MeOPhNHN)_2_CS, 1, was prepared from 2-methoxyaniline and ammonium sulfide according to the procedure reported by Mirkhalaf *et al.*, 1998. The synthesis of the title compound, *ortho*-methoxy dehydrodithizone, 2, was done according to a method by Irving *et al.*, (1971) as follows. A solution of (*o*-MeOPhNHN)_2_CS (0.2 g, 0.6 mmol) in dichloromethane (60 ml) was stirred (2 hrs) with a solution of potassium hexacyanoiron (III) (0.48 g) and potassium carbonate (0.46 g) in water (20 ml). The organic layer was removed, washed with water, and the solvent removed under reduced pressure. The product residue, on recrystallization from a minimum dichloromethane in acetone and water, gave 0.098 g orange-brown crystals of 2. Yield: 49%

Analytical data: *M*.p 192 ° C λ_max_(acetone) 445.6 nm (ε = 1360 dm^3^ mol^-1^ cm^-1^) δ_H_ (300 MHz, (CD_3_)_2_SO, 7.10 (1 H, t, *p*-C_6_H_5_), 7.21 (1 H, d, *m*-C_6_H_5_), 7.61 (1 H, t, *m*-C_6_H_5_), 7.76 (1 H, d, *o*-C_6_H_5_).

### Refinement

The aromatic, methylene and methyl H atoms were placed in geometrically idealized positions (C—H = 0.95 - 0.99 Å) and constrained to ride on their parent atoms with *U*_iso_(H) = 1.2*U*_eq_(C) for aromatic and methylene, and *U*_iso_(H) = 1.5*U*_eq_(C) for methyl protons. Torsion angles for methyl protons on the dehydrodithizone were refined from electron density, while those on the acetone solvent molecule as staggered. Large anisotropic displacements were observed on the proposed acetone solvent molecule which was subsequently treated as disordered. From this we were able to detect a minor component of dichloromethane solvate as well. The occupancy ratios for these two solvent molecules were obtained from free-refining their occupancies, and later fixing these values to 80:10 for acetone and dichloromethane, respectively. The positions of the –CH_3_ and –CH_2_ moieties of the two solvents could not be defined clearly and was subsequently refined as a fully occupied carbon site. The final result is a acetone molecule lying on a twofold rotation axis with the dichloromethane occupying two positions.

### Crystal data

**Table:**

C_15_H_14_N_4_O_2_S·0.4C_3_H_6_O·0.1CH_2_Cl_2_	*F*(000) = 1448
*M**_r_* = 346.09	*D*_x_ = 1.417 Mg m^−^^3^
Orthorhombic, *P**b**c**n*	Mo *K*α radiation, λ = 0.71073 Å
Hall symbol: -P 2n 2ab	Cell parameters from 1519 reflections
*a* = 19.5069 (13) Å	θ = 2.5–24.4°
*b* = 12.5245 (7) Å	µ = 0.25 mm^−^^1^
*c* = 13.278 (1) Å	*T* = 100 K
*V* = 3244.0 (4) Å^3^	Needle, red
*Z* = 8	0.33 × 0.12 × 0.11 mm

### Data collection

**Table:**

Bruker X8 APEXII 4K Kappa CCD diffractometer	4013 independent reflections
Radiation source: fine-focus sealed tube	2571 reflections with *I* > 2σ(*I*)
graphite	*R*_int_ = 0.051
Detector resolution: 8.4 pixels mm^-1^	θ_max_ = 28.3°, θ_min_ = 2.1°
φ and ω scans	*h* = −15→26
Absorption correction: multi-scan (*SADABS*; Bruker, 2008)	*k* = −12→16
*T*_min_ = 0.919, *T*_max_ = 0.972	*l* = −13→17
10890 measured reflections	

### Refinement

**Table:**

Refinement on *F*^2^	2 restraints
Least-squares matrix: full	H-atom parameters constrained
*R*[*F*^2^ > 2σ(*F*^2^)] = 0.059	*w* = 1/[σ^2^(*F*_o_^2^) + (0.0769*P*)^2^ + 1.158*P*] where *P* = (*F*_o_^2^ + 2*F*_c_^2^)/3
*wR*(*F*^2^) = 0.169	(Δ/σ)_max_ < 0.001
*S* = 1.06	Δρ_max_ = 0.84 e Å^−^^3^
4013 reflections	Δρ_min_ = −0.57 e Å^−^^3^
238 parameters	

### Special details

**Table:**

Experimental. The intensity data was collected on a Bruker X8 Apex II 4 K Kappa CCD diffractometer using an exposure time of 200 s/frame. A total of 358 frames were collected with a frame width of 0.5° covering up to θ = 28.30° with 99.2% completeness accomplished.
Geometry. All e.s.d.'s (except the e.s.d. in the dihedral angle between two l.s. planes) are estimated using the full covariance matrix. The cell e.s.d.'s are taken into account individually in the estimation of e.s.d.'s in distances, angles and torsion angles; correlations between e.s.d.'s in cell parameters are only used when they are defined by crystal symmetry. An approximate (isotropic) treatment of cell e.s.d.'s is used for estimating e.s.d.'s involving l.s. planes.

### Fractional atomic coordinates and isotropic or equivalent isotropic displacement parameters (Å^2^)

**Table:**

	*x*	*y*	*z*	*U*_iso_*/*U*_eq_	Occ. (<1)
S1	0.94113 (4)	0.35569 (6)	0.39602 (6)	0.0240 (2)	
N1	0.92670 (12)	0.20582 (17)	0.25103 (17)	0.0175 (5)	
N2	0.90050 (11)	0.20201 (16)	0.16004 (17)	0.0164 (5)	
N3	0.87217 (12)	0.29604 (17)	0.13628 (18)	0.0175 (5)	
N4	0.87976 (12)	0.36420 (17)	0.21135 (18)	0.0191 (5)	
C3	0.91500 (14)	0.3082 (2)	0.2832 (2)	0.0184 (6)	
C11	0.83626 (14)	0.3154 (2)	0.0438 (2)	0.0175 (6)	
C12	0.77732 (14)	0.2548 (2)	0.0246 (2)	0.0189 (6)	
C13	0.74450 (15)	0.2681 (2)	−0.0670 (2)	0.0222 (6)	
H13	0.7052	0.2265	−0.0828	0.027*	
C14	0.76917 (17)	0.3422 (2)	−0.1354 (2)	0.0258 (7)	
H14	0.7464	0.3511	−0.198	0.031*	
C15	0.82674 (16)	0.4038 (2)	−0.1141 (2)	0.0255 (7)	
H15	0.8424	0.4553	−0.1613	0.031*	
C16	0.86101 (15)	0.3897 (2)	−0.0240 (2)	0.0220 (6)	
H16	0.9008	0.4303	−0.0089	0.026*	
O1	0.84463 (11)	0.01383 (15)	0.21656 (16)	0.0255 (5)	
C1	0.81522 (18)	−0.0853 (2)	0.2495 (3)	0.0347 (8)	
H1A	0.7779	−0.1056	0.204	0.052*	
H1B	0.7972	−0.0769	0.318	0.052*	
H1C	0.8505	−0.141	0.2491	0.052*	
C21	0.90302 (14)	0.1126 (2)	0.0934 (2)	0.0183 (6)	
C22	0.87405 (15)	0.0159 (2)	0.1243 (2)	0.0214 (6)	
C23	0.87648 (16)	−0.0689 (2)	0.0568 (2)	0.0274 (7)	
H23	0.8586	−0.1364	0.076	0.033*	
C24	0.90459 (16)	−0.0560 (2)	−0.0376 (3)	0.0299 (7)	
H24	0.9045	−0.1145	−0.0831	0.036*	
C25	0.93295 (16)	0.0403 (3)	−0.0676 (2)	0.0286 (7)	
H25	0.9524	0.0478	−0.1329	0.034*	
C26	0.93252 (15)	0.1258 (2)	−0.0009 (2)	0.0229 (6)	
H26	0.9522	0.1923	−0.0195	0.027*	
O2	0.75650 (10)	0.18958 (15)	0.10072 (14)	0.0218 (5)	
C2	0.71273 (16)	0.1018 (2)	0.0731 (3)	0.0298 (7)	
H2A	0.6689	0.1295	0.0483	0.045*	
H2B	0.7047	0.0565	0.1321	0.045*	
H2C	0.7348	0.0596	0.0201	0.045*	
C01	0.96482 (19)	0.3561 (3)	0.6608 (3)	0.0505 (13)	0.9
H02A	0.9476	0.299	0.6172	0.076*	0.8
H02B	0.9974	0.4004	0.6231	0.076*	0.8
H02C	0.9264	0.4004	0.6835	0.076*	0.8
H02D	0.9694	0.3499	0.5868	0.061*	0.1
H02E	0.9167	0.3784	0.672	0.061*	0.1
O01	1	0.2112 (4)	0.75	0.084 (2)	0.8
C02	1	0.3083 (5)	0.75	0.0332 (14)	0.8
Cl1	0.9637 (7)	0.2275 (7)	0.6975 (8)	0.061 (3)	0.1
Cl2	1.0037 (4)	0.4567 (5)	0.6826 (5)	0.0276 (16)	0.1

### Atomic displacement parameters (Å^2^)

**Table:**

	*U*^11^	*U*^22^	*U*^33^	*U*^12^	*U*^13^	*U*^23^
S1	0.0222 (4)	0.0265 (4)	0.0233 (4)	0.0002 (3)	−0.0020 (3)	−0.0080 (3)
N1	0.0164 (12)	0.0182 (11)	0.0179 (11)	−0.0017 (9)	−0.0018 (9)	0.0005 (10)
N2	0.0148 (12)	0.0151 (11)	0.0194 (12)	−0.0002 (9)	−0.0012 (9)	−0.0003 (10)
N3	0.0151 (12)	0.0147 (11)	0.0227 (12)	−0.0005 (9)	−0.0010 (10)	0.0011 (10)
N4	0.0180 (12)	0.0179 (11)	0.0213 (12)	−0.0017 (9)	−0.0009 (10)	−0.0045 (10)
C3	0.0148 (14)	0.0178 (13)	0.0225 (14)	−0.0017 (11)	0.0030 (11)	−0.0013 (12)
C11	0.0172 (14)	0.0171 (13)	0.0182 (13)	0.0041 (11)	−0.0004 (11)	−0.0006 (11)
C12	0.0174 (14)	0.0169 (13)	0.0224 (14)	0.0056 (11)	0.0021 (11)	−0.0008 (12)
C13	0.0197 (15)	0.0240 (15)	0.0230 (14)	0.0035 (12)	−0.0022 (12)	−0.0038 (13)
C14	0.0317 (18)	0.0259 (15)	0.0198 (14)	0.0060 (13)	−0.0029 (13)	−0.0002 (13)
C15	0.0310 (17)	0.0220 (14)	0.0236 (15)	0.0038 (13)	0.0024 (13)	0.0033 (13)
C16	0.0220 (15)	0.0184 (14)	0.0254 (15)	0.0004 (11)	0.0010 (12)	0.0006 (13)
O1	0.0309 (12)	0.0187 (10)	0.0268 (11)	−0.0068 (9)	−0.0002 (9)	0.0021 (9)
C1	0.041 (2)	0.0239 (16)	0.0396 (19)	−0.0137 (14)	−0.0026 (16)	0.0044 (15)
C21	0.0172 (14)	0.0146 (12)	0.0231 (14)	0.0036 (10)	−0.0048 (11)	−0.0048 (11)
C22	0.0185 (14)	0.0206 (14)	0.0250 (15)	0.0023 (11)	−0.0044 (12)	−0.0013 (12)
C23	0.0269 (17)	0.0186 (14)	0.0369 (18)	−0.0005 (12)	−0.0074 (14)	−0.0055 (14)
C24	0.0287 (17)	0.0282 (16)	0.0329 (17)	0.0085 (13)	−0.0108 (14)	−0.0107 (15)
C25	0.0264 (17)	0.0345 (17)	0.0249 (15)	0.0118 (13)	−0.0050 (13)	−0.0075 (15)
C26	0.0228 (16)	0.0236 (15)	0.0222 (14)	0.0064 (12)	−0.0031 (12)	−0.0011 (12)
O2	0.0205 (11)	0.0231 (10)	0.0219 (10)	−0.0053 (8)	0.0001 (9)	0.0014 (9)
C2	0.0250 (17)	0.0301 (16)	0.0342 (17)	−0.0101 (13)	−0.0068 (14)	0.0055 (15)
C01	0.0178 (18)	0.077 (3)	0.057 (3)	0.003 (2)	−0.0090 (19)	0.048 (2)
O01	0.140 (7)	0.026 (3)	0.085 (5)	0	−0.037 (5)	0
C02	0.041 (4)	0.033 (3)	0.026 (3)	0	−0.005 (3)	0
Cl1	0.087 (9)	0.052 (6)	0.043 (6)	−0.052 (6)	0.001 (6)	−0.002 (5)
Cl2	0.031 (4)	0.031 (4)	0.021 (3)	0.016 (3)	0.005 (3)	0.002 (3)

### Geometric parameters (Å, °)

**Table:**

S1—C3	1.690 (3)	C21—C26	1.387 (4)
N1—N2	1.313 (3)	C21—C22	1.398 (4)
N1—C3	1.371 (3)	C22—C23	1.391 (4)
N2—N3	1.339 (3)	C23—C24	1.377 (5)
N2—C21	1.428 (3)	C23—H23	0.95
N3—N4	1.321 (3)	C24—C25	1.386 (5)
N3—C11	1.434 (4)	C24—H24	0.95
N4—C3	1.369 (3)	C25—C26	1.390 (4)
C11—C16	1.382 (4)	C25—H25	0.95
C11—C12	1.401 (4)	C26—H26	0.95
C12—O2	1.361 (3)	O2—C2	1.439 (3)
C12—C13	1.385 (4)	C2—H2A	0.98
C13—C14	1.385 (4)	C2—H2B	0.98
C13—H13	0.95	C2—H2C	0.98
C14—C15	1.392 (4)	C01—C02	1.494 (4)
C14—H14	0.95	C01—Cl2	1.499 (7)
C15—C16	1.382 (4)	C01—Cl1	1.683 (8)
C15—H15	0.95	C01—H02A	0.98
C16—H16	0.95	C01—H02B	0.98
O1—C22	1.353 (3)	C01—H02C	0.98
O1—C1	1.436 (3)	C01—H02D	0.99
C1—H1A	0.98	C01—H02E	0.99
C1—H1B	0.98	O01—C02	1.216 (8)
C1—H1C	0.98	C02—C01^i^	1.494 (4)
			
Cg···Cg^ii^	3.886 (3)		
			
N2—N1—C3	104.8 (2)	C23—C24—H24	119.2
N1—N2—N3	110.2 (2)	C25—C24—H24	119.2
N1—N2—C21	125.9 (2)	C24—C25—C26	119.0 (3)
N3—N2—C21	123.9 (2)	C24—C25—H25	120.5
N4—N3—N2	110.2 (2)	C26—C25—H25	120.5
N4—N3—C11	126.3 (2)	C21—C26—C25	119.1 (3)
N2—N3—C11	123.5 (2)	C21—C26—H26	120.4
N3—N4—C3	104.6 (2)	C25—C26—H26	120.4
N4—C3—N1	110.2 (2)	C12—O2—C2	116.5 (2)
N4—C3—S1	126.1 (2)	O2—C2—H2A	109.5
N1—C3—S1	123.7 (2)	O2—C2—H2B	109.5
C16—C11—C12	122.2 (3)	H2A—C2—H2B	109.5
C16—C11—N3	120.1 (2)	O2—C2—H2C	109.5
C12—C11—N3	117.7 (2)	H2A—C2—H2C	109.5
O2—C12—C13	125.9 (3)	H2B—C2—H2C	109.5
O2—C12—C11	115.8 (2)	C02—C01—Cl2	87.2 (4)
C13—C12—C11	118.3 (3)	C02—C01—Cl1	52.7 (4)
C12—C13—C14	119.7 (3)	Cl2—C01—Cl1	139.1 (5)
C12—C13—H13	120.1	C02—C01—H02A	109.5
C14—C13—H13	120.1	Cl2—C01—H02A	154.4
C13—C14—C15	121.2 (3)	Cl1—C01—H02A	57.8
C13—C14—H14	119.4	C02—C01—H02B	109.5
C15—C14—H14	119.4	Cl2—C01—H02B	45.2
C16—C15—C14	119.7 (3)	Cl1—C01—H02B	134.2
C16—C15—H15	120.2	H02A—C01—H02B	109.5
C14—C15—H15	120.2	C02—C01—H02C	109.5
C15—C16—C11	118.8 (3)	Cl2—C01—H02C	81.5
C15—C16—H16	120.6	Cl1—C01—H02C	116.2
C11—C16—H16	120.6	H02A—C01—H02C	109.5
C22—O1—C1	117.5 (2)	H02B—C01—H02C	109.5
O1—C1—H1A	109.5	C02—C01—H02D	135.6
O1—C1—H1B	109.5	Cl2—C01—H02D	102.3
H1A—C1—H1B	109.5	Cl1—C01—H02D	102.3
O1—C1—H1C	109.5	H02A—C01—H02D	52.1
H1A—C1—H1C	109.5	H02B—C01—H02D	58.7
H1B—C1—H1C	109.5	H02C—C01—H02D	114.8
C26—C21—C22	122.4 (3)	C02—C01—H02E	115.4
C26—C21—N2	118.7 (2)	Cl2—C01—H02E	102.3
C22—C21—N2	118.9 (3)	Cl1—C01—H02E	102.3
O1—C22—C23	125.7 (3)	H02A—C01—H02E	88.3
O1—C22—C21	117.0 (2)	H02B—C01—H02E	122.1
C23—C22—C21	117.3 (3)	H02C—C01—H02E	21.5
C24—C23—C22	120.7 (3)	H02D—C01—H02E	104.9
C24—C23—H23	119.7	O01—C02—C01^i^	113.6 (3)
C22—C23—H23	119.7	O01—C02—C01	113.6 (3)
C23—C24—C25	121.5 (3)	C01^i^—C02—C01	132.8 (6)
			
C3—N1—N2—N3	−1.4 (3)	C12—C11—C16—C15	−0.6 (4)
C3—N1—N2—C21	176.8 (2)	N3—C11—C16—C15	177.6 (2)
N1—N2—N3—N4	0.5 (3)	N1—N2—C21—C26	−122.6 (3)
C21—N2—N3—N4	−177.7 (2)	N3—N2—C21—C26	55.4 (4)
N1—N2—N3—C11	−176.7 (2)	N1—N2—C21—C22	59.1 (4)
C21—N2—N3—C11	5.1 (4)	N3—N2—C21—C22	−122.9 (3)
N2—N3—N4—C3	0.6 (3)	C1—O1—C22—C23	2.2 (4)
C11—N3—N4—C3	177.7 (2)	C1—O1—C22—C21	−179.5 (3)
N3—N4—C3—N1	−1.5 (3)	C26—C21—C22—O1	−177.7 (3)
N3—N4—C3—S1	178.6 (2)	N2—C21—C22—O1	0.5 (4)
N2—N1—C3—N4	1.8 (3)	C26—C21—C22—C23	0.8 (4)
N2—N1—C3—S1	−178.2 (2)	N2—C21—C22—C23	179.0 (2)
N4—N3—C11—C16	66.4 (4)	O1—C22—C23—C24	176.4 (3)
N2—N3—C11—C16	−116.8 (3)	C21—C22—C23—C24	−1.9 (4)
N4—N3—C11—C12	−115.2 (3)	C22—C23—C24—C25	1.8 (5)
N2—N3—C11—C12	61.6 (3)	C23—C24—C25—C26	−0.4 (4)
C16—C11—C12—O2	−176.0 (2)	C22—C21—C26—C25	0.6 (4)
N3—C11—C12—O2	5.6 (4)	N2—C21—C26—C25	−177.7 (2)
C16—C11—C12—C13	2.1 (4)	C24—C25—C26—C21	−0.8 (4)
N3—C11—C12—C13	−176.2 (2)	C13—C12—O2—C2	22.9 (4)
O2—C12—C13—C14	176.1 (3)	C11—C12—O2—C2	−159.1 (3)
C11—C12—C13—C14	−1.9 (4)	Cl2—C01—C02—O01	−160.1 (3)
C12—C13—C14—C15	0.2 (4)	Cl1—C01—C02—O01	11.4 (6)
C13—C14—C15—C16	1.3 (5)	Cl2—C01—C02—C01^i^	19.9 (3)
C14—C15—C16—C11	−1.1 (4)	Cl1—C01—C02—C01^i^	−168.6 (6)

Symmetry codes: (i) −*x*+2, *y*, −*z*+3/2; (ii) −*x*+2, −*y*, −*z*.
